# Switch in *KRAS* mutational status during an unusual course of disease in a patient with advanced pancreatic adenocarcinoma: implications for translational research

**DOI:** 10.1186/s12885-017-3376-4

**Published:** 2017-05-26

**Authors:** Sibylle Baechmann, Steffen Ormanns, Michael Haas, Stephan Kruger, Anna Remold, Dominik Paul Modest, Thomas Kirchner, Andreas Jung, Jens Werner, Volker Heinemann, Stefan Boeck

**Affiliations:** 1 Institute of Pathology, Ludwig-Maximilians University of Munich, Munich, Germany; 2 Department of Internal Medicine III and Comprehensive Cancer Center, Klinikum Grosshadern, Ludwig-Maximilians University of Munich, Marchioninistr. 15, 81377 Munich, Germany; 3 Department of General, Visceral, Vascular and Transplantation Surgery, Klinikum Grosshadern, Ludwig-Maximilians-University of Munich, Munich, Germany; 4 DKTK, German Cancer Consortium, German Cancer Research Center (DKFZ), Heidelberg, Germany

**Keywords:** Pancreatic ductal adenocarcinoma (PDAC), *KRAS* mutation, Tumor heterogeneity

## Abstract

**Background:**

Despite the introduction of novel effective treatment regimens like gemcitabine plus nab-paclitaxel and FOLFIRINOX, pancreatic ductal adenocarcinoma (PDAC) remains one of the most aggressive epithelial tumors. Among the genetic alterations frequently found in PDAC, mutations in the *KRAS* gene might play a prognostic role regarding overall survival and may also have the potential to predict the efficacy of anti-EGFR treatment.

**Case presentation:**

We report the clinical case of a 69 year old Caucasian female that was diagnosed with histologically confirmed locally advanced PDAC with lymph node involvement in August 2010. At the time of first diagnosis, tumor tissue obtained from an open regional lymph node biopsy showed a poorly differentiated adenocarcinoma with a wild type sequence within exon 2 (codon 12/13) of the *KRAS* gene. The patient initially received single-agent gemcitabine and a subsequent 5-FU-based chemoradiotherapy with a sequential maintenance chemotherapy with oral capecitabine resulting in a long term disease control. Local disease progression occurred in May 2014 and the patient underwent pancreaticoduodenectomy in September 2014. A novel *KRAS* gene mutation (c.35G > T, p.G12 V) in exon 2 (codon 12) was detected within the surgical specimen. As of January 2016 the patient is still alive and without evidence of the underlying disease.

**Conclusions:**

Specifically in the context of clinical trials and translational research in PDAC a re-assessment of molecular biomarkers, i. e. *KRAS*, at defined time points (e. g. relapse, disease progression, unusual clinical course) may be indicated in order to detect a potential switch in biomarker status during the course of disease.

## Background

Pancreatic ductal adenocarcinoma (PDAC) is one of the most aggressive epithelial tumors worldwide. In most patients it represents a deadly disease [1] due to an advanced stage at the time of diagnosis and the difficulties in therapeutic treatment, but also due to genetic heterogeneity [2]. Surgical resection remains the only curative treatment option for localized PDAC. During the last decade, systemic treatment with single-agent gemcitabine has evolved as standard chemotherapy for the adjuvant and palliative treatment setting [3, 4]. Gemcitabine offers a median survival of about 5 to 7 months in patients with advanced disease and shows comparatively good tolerability [5]; more recently, gemcitabine-based combination regimens with the oral epidermal growth factor receptor (EGFR) inhibitor erlotinib or together with nab-paclitaxel [6] showed a statistically significant improvement in overall survival (OS). The development and progression of PDAC include different genetic alterations in oncogenic activation, loss of tumor-suppressor gene function and overexpression of receptor-ligand systems [7, 8]. Among these genetic alterations, mutations in the *KRAS* gene, which often are already present in precursor lesions, play an important role in tumor development and progression [8]. Gain of function mutations in the *KRAS* gene are detected in about 70 to 90% of PDAC cases [9], commonly as point mutations in exon 2 (codon 12/13), most frequently as p.G12D (c.35G > A) or p.G12 V (p.35G > T). Several studies showed that constitutively activating *KRAS* mutations are associated with worse OS, whereas *KRAS* wildtype status is associated with improved OS in PDAC [7, 10, 11]. Thus, in PDAC, *KRAS* mutations may be regarded as prognostic biomarker. The role of *KRAS* mutational status as predictive biomarker regarding the use of EGFR-targeting agents like erlotinib in advanced PDAC still remains a matter of debate to date [12–14].

Here, we report the case of a PDAC patient with an unusual clinical course: the tumor of the patient harbored a wildtype *KRAS* gene at the time of initial PDAC diagnosis; however, upon disease progression 4 years later, a mutation within exon 2 of the *KRAS* gene was detectable.

## Case presentation

A currently 75-year-old woman was diagnosed with locally advanced PDAC at our comprehensive cancer center (CCC) in 2010. An explorative laparotomy in August 2010 showed metastatic disease spread extensively to regional lymph nodes and thus the primary tumor in the pancreatic head was not resected. By CT imaging criteria no other distant metastatic disease was evident. Lymph nodes were sampled surgically from the right gastric artery, the hepatic artery, the coeliac trunc and from the interaortocaval region; in all samples, tumor infiltration by a poorly differentiated adenocarcinoma was confirmed by histology. Immunohistochemical staining was positive for CK7, CK20 and CA 19–9 (with CDX-2 being negative). At that time point an additional analysis for *KRAS* mutational status and EGFR protein expression (which were conducted within a translational research project) detected a wildtype sequence of *KRAS* exon 2 by pyrosequencing and a moderately positive immunohistochemical staining for membranous EGFR expression in about 80% of the tumor cells.

**Fig. 1:**
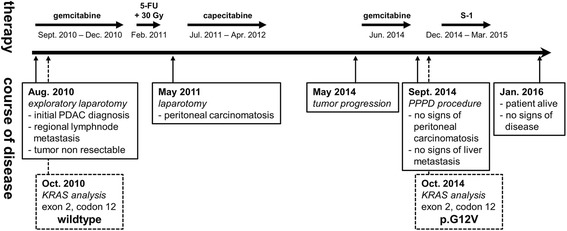
Therapy, procedures and *KRAS* mutational status over the time course of the disease (5-FU = 5-fluouracile, Gy = Gray, PPPD = pylorus preserving pancreaticoduodenectomy)

The patient initially received systemic chemotherapy with three cycles of standard dose (1000 mg/m^2^) gemcitabine between September and December 2010. Imaging studies in January 2011 confirmed stable disease and the CA 19–9 levels decreased from 3700 U/ml at first diagnosis to 180 U/ml. In February 2011 5-FU-based chemoradiotherapy (30 Gy) was applied at an external hospital. During re-exploration performed in May 2011 surgical biopsies from the peritoneum histologically confirmed metastatic disease of PDAC; thus, no attempt to resect the primary tumor in the pancreas was performed. We then decided, also based on the wish of the patient, to re-start systemic chemotherapy and treatment with oral capecitabine was initiated in July 2011 and given until April 2012. During this chemotherapy, a further decline of CA 19–9 values was observed (nadir: 30 U/ml) and repeated CT imaging did not show any signs of local disease progression or metastatic disease (as assessed by imaging criteria). After a treatment rest for two years (beginning in May 2012), local tumor progression of the pancreatic primary was observed within a CT scan in May 2014. Again, no radiographic signs of distant metastasis were observed. Systemic chemotherapy with single-agent gemcitabine was re-introduced in June 2014 resulting in a CA 19–9 decrease from 690 U/ml at disease progression to 380 U/ml after three gemcitabine applications. Due to a progressive duodenal infiltration with clinical and endoscopic signs of gastrointestinal obstruction, a surgical re-exploration was performed in September 2014. Intraoperatively, no signs of peritoneal carcinomatosis were apparent and a liver biopsy showed no signs of malignancy. Thus, the pancreatic primary was removed by a pylorus preserving pancreaticoduodenectomy (modified Whipple-Kausch procedure). The tumor was classified as ypT3 ypN0 (0/15) L0 V0 Pn0, ductal adenocarcinoma G3, R0 resection (according to UICC criteria, TNM classification 7th edition, 2010). An additionally executed *KRAS* mutational analysis at this time point revealed a new point mutation p.G12 V (c.35G > T) in exon 2, codon 12. After surgery, CA 19–9 values decreased to levels of 20 U/ml. The patient was offered adjuvant chemotherapy with S-1 (tegafur, gimeracil, oteracil) after pancreaticoduodenectomy and started this treatment December 2014; however, S-1 was tolerated poorly due to gastrointestinal toxicity (diarrhea grade 4 and accompanying renal insufficiency) and was therefore terminated in March 2015. As of January 2016 the patient is still alive and without clear evidence of the underlying disease. An overview of this unusual disease course is shown within Fig. 1.

## Discussion

Up to now, no prognostic or predictive tissue biomarker is available for PDAC [12]. In contrast to other diseases like breast, lung or colorectal cancer no specific biomarker has been validated for clinical use in pancreatic cancer and several clinical and translational trials are ongoing in order to better define the molecular basis of this disease and to search specifically for predictive markers for treatment efficacy. Thus, only limited data is available on the clinical role of biomarkers in PDAC [12]; specifically, there are no clear recommendations at which time points biomarkers should be assessed. In CRC for example, a good correlation between biomarker results from the primary tumor and from (metachronous) CRC metastases has been reported, resulting in the acceptance of e. g. *RAS* status of primary tumor tissue in patients with a metachronous relapse [15]. In contrast, in other diseases like breast cancer a switch in e. g. Her2/neu (ERBB2) status is well known resulting in the recommendation of repeated tumor biopsies at relapse or disease progression [16]. At least to our knowledge, studies investigating this issue have not yet been performed in PDAC.

Within this manuscript we report a rather unusual clinical course of a PADC patient, with a corresponding switch in *KRAS* mutational status during the course of disease. Of note, we detected the new *KRAS* mutation upon disease progression in September 2014; furthermore, it may be important to highlight the fact that this patient did not receive previous anti-EGFR treatment (e.g. with erlotinib) before the detection of the new *KRAS* mutation.

**Fig. 2:**
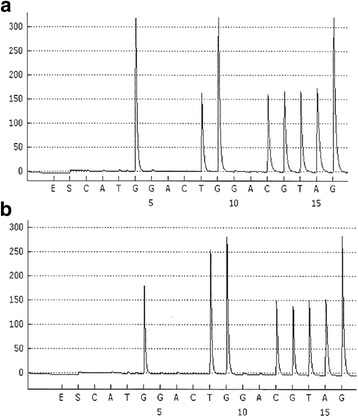
Pyrograms comparing the tumors *KRAS* exon 2, codon 12 mutational status in **a** October 2010 (wildtype sequence GGTGGC) and **b** October 2014 (point mutation p.G12 V, c. 35 G > T, sequence GTTGGC)

Several possible explanations may be hypothesized for the observation of a *KRAS* switch during the course of disease in our PDAC patient:

1. Appearance of a truly new tumor KRAS mutation upon disease progression in September 2014 without previous application of agents targeting the EGFR pathway:
  The reason for tumor progression could be caused by an evolved new mutation event in the *KRAS* gene, specifically in the light of selection pressure during previous treatment with chemotherapy and radiotherapy. In colorectal cancer, increasing evidence exists that the appearance of new *KRAS* mutations during treatment with agents targeting the EGFR (like cetuximab or panitumumab) may be linked to an acquired resistance to anti-EGFR therapy [17, 18]. Of note, our patient did not receive anti-EGFR treatment for example with erlotinib before the detection of the new *KRAS* mutation. If other treatments like cytotoxic chemotherapy (gemcitabine, fluoropyrimidines) or radiotherapy to the pancreatic primary may also induce a “selection pressure” for the development of new genetic events remains unknown.
2. Tumor heterogeneity with distinct results in KRAS analysis at initial diagnosis (lymph node metastasis analyzed) and at progression (primary tumor analyzed):
  There is increasing evidence for intratumoral heterogeneity in different types of cancer that could be determined by multiregion sequencing [19]. In non-small cell lung cancer it was shown that ALK rearrangements (that were previously thought to be mutually exclusive with activating *EGFR* and *KRAS* mutations) can be found together with EGFR mutations in rare cases [20]. Moreover, it was shown that spatially separated subclones of the same tumor harbor different oncogenic drivers [21]. If these observations are transferable to PDAC, this might explain the differences in *KRAS* mutational status observed in our patient reported here. However, the scarce currently available data comparing pancreatic primary tumors and corresponding metastases, showed the same *KRAS* mutational status in the primary tumor and each metastatic site examined, thus supporting the idea of a newly apparent KRAS mutation [22, 23].
3. Technical aspects of the discrepant KRAS sequencing results (see Fig. 2):
  Potentially, the initial *KRAS* wildtype status detected in 2010 could be the effect of a false negative sequencing result. Both *KRAS* analyses in the tumor tissue of the patient reported here were performed in the same specialized and certified laboratory for molecular pathology. For both analyses, formalin fixed paraffin embedded (FFPE) tumor tissue was microdissected under visual control using a microscope to reduce contamination by adjacent normal tissue. In both situations, sufficient tumor tissue was available: In 2010 a subtotally infiltrated lymph node metastasis, 22 mm in diameter, containing insignificant residual lymphatic tissue and in 2014 whole tumor resection tissue was used for analysis. Moreover, the pyrosequencing assay employed here is highly sensitive and requires only 10% of tumor DNA in the whole DNA extracted to reliably detect the *KRAS* mutational status [24]. Thus, a false negative sequencing result is a very unlikely event to explain the discrepancy in the present case.

## Conclusions

*KRAS* mutational status may change during the course of disease in PDAC. Thus, in well-defined clinical scenarios (e. g. relapse after surgery in curative intent, disease progression during/after chemotherapy, unusual clinical course) a re-assessment of the *KRAS* status should be discussed, specifically within the setting of controlled clinical and translational trials. As *KRAS* is not yet established as a clinically relevant biomarker in PDAC, future translational trials in pancreatic cancer that evaluate a broad range of novel biomarkers should, at least to our opinion, include a repeated biomarker assessment during the course of disease within their prospective study protocols. Novel promising techniques like liquid biopsy approaches may thereby help to overcome the limitations of obtaining tumor tissue safely in PDAC [25]. As it may be difficult to obtain sufficient tumor tissue in PDAC by percutaneous- or endosonography-guided biopsy techniques, a sampling error may occur specifically in the light of tumor heterogeneity. In that context, liquid biopsy techniques may also eventually help to overcome these limitations.

## Abbreviations

5-FU: 5-fluorouracil
ALK: Anaplastic lymphoma kinase
CA19–9: Carbohydrate antigen 19–9
CDX2: Caudal type homeo-box transcription factor 2
CK: Cytokeratin
CRC: Colorectal cancer
CT: Computed tomography
EGFR: Epidermal growth factor receptor
ERBB2: Human epidermal growth factor receptor 2
FFPE: Formalin fixed paraffin embedded
FOLFIRINOX: Folinic acid, 5-FU, irinotecan, oxaliplatin
Gy: Gray
KRAS: Kirsten rat sarcoma viral oncogene homologue
nab-paclitaxel: Nanoparticle albumin-bound paclitaxel
OS: Overall survival
PDAC: Pancreatic ductal adenocarcinoma
UICC: International union against cancer
